# Supplementary material for: Anti-inflammatory and immunomodulatory therapies are associated with reduced risk of age-associated neurodegenerative diseases: impact of sex and treatment duration
Source: Front Aging Neurosci. 2026 May 18;18:1818660. doi: 10.3389/fnagi.2026.1818660 (PMC13223144; doi:10.3389/fnagi.2026.1818660)
Supplement: Supplementary file 1 [file Data_Sheet_1.docx]

| **Table S1. List of Drug Codes Used** | |
| --- | --- |
| **Drug** | **Code for Drugs** |
| Antidiabetics | GENERIC_DRUG-METFORMIN_HCL, GENERIC_DRUG-GLYBURIDE, GENERIC_DRUG-GLYBURIDE_MICRONIZED, GENERIC_DRUG-GLIMEPIRIDE, GENERIC_DRUG-GLIPIZIDE, GENERIC_DRUG-TOLAZAMIDE, GENERIC_DRUG-TOLBUTAMIDE, GENERIC_DRUG-CHLORPROPAMIDE, GENERIC_DRUG-PIOGLITAZONE_HCL, GENERIC_DRUG-ALOGLIPTIN_BENZ/PIOGLITAZONE, GENERIC_DRUG-PIOGLITAZONE_HCL/GLIMEPIRIDE, GENERIC_DRUG-PIOGLITAZONE_HCL/METFORMIN_HCL, GENERIC_DRUG-ROSIGLITAZONE/GLIMEPIRIDE, GENERIC_DRUG-ROSIGLITAZONE/METFORMIN_HCL, GENERIC_DRUG-ROSIGLITAZONE_MALEATE, GENERIC_DRUG-ALOGLIPTIN_BENZ/METFORMIN_HCL, GENERIC_DRUG-CANAGLIFLOZIN/METFORMIN_HCL, GENERIC_DRUG-DAPAGLIFLOZIN/METFORMIN_HCL, GENERIC_DRUG-EMPAGLIFLOZIN/METFORMIN_HCL, GENERIC_DRUG-ERTUGLIFLOZIN/METFORMIN, GENERIC_DRUG-GLIPIZIDE/METFORMIN_HCL, GENERIC_DRUG-GLYBURIDE/METFORMIN_HCL, GENERIC_DRUG-GLYBURIDE__MICRO/METFORMIN_HCL, GENERIC_DRUG-LINAGLIPTIN/METFORMIN_HCL, GENERIC_DRUG-METFORMIN/CAFF/AA7/HRB125/CHOL, GENERIC_DRUG-REPAGLINIDE/METFORMIN_HCL, GENERIC_DRUG-SAXAGLIPTIN_HCL/METFORMIN_HCL, GENERIC_DRUG-SITAGLIPTIN_PHOS/METFORMIN_HCL, GENERIC_DRUG-NATEGLINIDE, GENERIC_DRUG-REPAGLINIDE, GENERIC_DRUG-ACARBOSE, GENERIC_DRUG-MIGLITOL, GENERIC_DRUG-CANAGLIFLOZIN, GENERIC_DRUG-DAPAGLIFLOZIN/SAXAGLIPTIN_HCL, GENERIC_DRUG-DAPAGLIFLOZIN_PROPANEDIOL, GENERIC_DRUG-EMPAGLIFLOZIN, GENERIC_DRUG-EMPAGLIFLOZIN/LINAGLIPTIN, GENERIC_DRUG-ERTUGLIFLOZIN/SITAGLIPTIN, GENERIC_DRUG-ERTUGLIFLOZIN_PIDOLATE, GENERIC_DRUG-EXENATIDE, GENERIC_DRUG-EXENATIDE_MICROSPHERES, GENERIC_DRUG-LIXISENATIDE, GENERIC_DRUG-ALBIGLUTIDE, GENERIC_DRUG-DULAGLUTIDE, GENERIC_DRUG-LIRAGLUTIDE, GENERIC_DRUG-SEMAGLUTIDE, GENERIC_DRUG-TEDUGLUTIDE, GENERIC_DRUG-ALOGLIPTIN_BENZOATE, GENERIC_DRUG-LINAGLIPTIN, GENERIC_DRUG-SAXAGLIPTIN_HCL, GENERIC_DRUG-SITAGLIPTIN/SIMVASTATIN, GENERIC_DRUG-SITAGLIPTIN_PHOSPHATE, GENERIC_DRUG-HUM_INSULIN_NPH/REG_INSULIN_HM, GENERIC_DRUG-INFUSION_SET-INSULIN_PUMP_BODY, GENERIC_DRUG-INFUSION_SET_FOR_INSULIN_PUMP, GENERIC_DRUG-INSULIN_ADMIN._SUPPLIES, GENERIC_DRUG-INSULIN_ASPART, GENERIC_DRUG-INSULIN_ASPART_(NIACINAMIDE), GENERIC_DRUG-INSULIN_ASPART_PROT/INSULN_ASP, GENERIC_DRUG-INSULIN_ASPART_PROTAM_&_ASPART, GENERIC_DRUG-INSULIN_DEGLUDEC, GENERIC_DRUG-INSULIN_DEGLUDEC/LIRAGLUTIDE, GENERIC_DRUG-INSULIN_DETEMIR, GENERIC_DRUG-INSULIN_GLARGINE/LIXISENATIDE, GENERIC_DRUG-INSULIN_GLARGINE_HUM.REC.ANLOG, GENERIC_DRUG-INSULIN_GLULISINE, GENERIC_DRUG-INSULIN_ISOPHANE_NPH_BF-PK, GENERIC_DRUG-INSULIN_LISPRO, GENERIC_DRUG-INSULIN_LISPRO_PROTAMIN/LISPRO, GENERIC_DRUG-INSULIN_NPH_HUM/REG_INSULIN_HM, GENERIC_DRUG-INSULIN_NPH_HUMAN_ISOPHANE, GENERIC_DRUG-INSULIN_NPL/INSULIN_LISPRO, GENERIC_DRUG-INSULIN_PUMP/INFUS._SET/METER, GENERIC_DRUG-INSULIN_PUMP_CARTRIDGE, GENERIC_DRUG-INSULIN_PUMP_CONTROLLER, GENERIC_DRUG-INSULIN_PUMP_SYRINGE__1.8_ML, GENERIC_DRUG-INSULIN_PUMP_SYRINGE__3_ML, GENERIC_DRUG-INSULIN_REGULAR_BEEF-PORK, GENERIC_DRUG-INSULIN_REGULAR__HUMAN, GENERIC_DRUG-INSULIN_ULTRALENTE, GENERIC_DRUG-INSULIN_ZINC_BEEF-PORK, GENERIC_DRUG-INSULN_ASP_PRT/INSULIN_ASPART, GENERIC_DRUG-NEEDLELESS_ACCESS._DEV_INSULIN, GENERIC_DRUG-NEEDLES__INSULIN_DISP.__SAFETY, GENERIC_DRUG-NEEDLES__INSULIN_DISPOSABLE, GENERIC_DRUG-NPH__HUMAN_INSULIN_ISOPHANE, GENERIC_DRUG-SUB-Q_INSULIN_DEVICE__20_UNIT, GENERIC_DRUG-SUB-Q_INSULIN_DEVICE__30_UNIT, GENERIC_DRUG-SUB-Q_INSULIN_DEVICE__40_UNIT, GENERIC_DRUG-SUBCUTANEOUS_INSULIN_PUMP, GENERIC_DRUG-SUBQ_INSULIN_PUMP_GLUC.MON.SYS, GENERIC_DRUG-SYRINGE-NEEDLE_INSULIN_0.5_ML, GENERIC_DRUG-SYRINGE_&_NEEDLE_INSULIN_1_ML, GENERIC_DRUG-SYRINGE_AND_NEEDLE_INSULIN_1ML, GENERIC_DRUG-SYRINGE_INSULIN_NEEDLESS_1_ML, GENERIC_DRUG-SYRINGE_W-NDL__DISP.__INSULIN, GENERIC_DRUG-SYRINGE_WITH_NEEDLE__INSULIN, GENERIC_DRUG-SYR_NDL_INSULIN_1ML-SHARPS_BIN |
| Antihypertensives | GENERIC_DRUG-LISINOPRIL, GENERIC_DRUG-RAMIPRIL, GENERIC_DRUG-PERINDOPRIL_ERBUMINE, GENERIC_DRUG-QUINAPRIL_HCL, GENERIC_DRUG-CAPTOPRIL, GENERIC_DRUG-MOEXIPRIL_HCL, GENERIC_DRUG-TRANDOLAPRIL, GENERIC_DRUG-ENALAPRILAT_DIHYDRATE, GENERIC_DRUG-ENALAPRIL_MALEATE, GENERIC_DRUG-LISINOPRIL/HYDROCHLOROTHIAZIDE, GENERIC_DRUG-QUINAPRIL/HYDROCHLOROTHIAZIDE, GENERIC_DRUG-CAPTOPRIL/HYDROCHLOROTHIAZIDE, GENERIC_DRUG-MOEXIPRIL/HYDROCHLOROTHIAZIDE, GENERIC_DRUG-ENALAPRIL/HYDROCHLOROTHIAZIDE, GENERIC_DRUG-LOSARTAN_POTASSIUM, GENERIC_DRUG-VALSARTAN, GENERIC_DRUG-IRBESARTAN, GENERIC_DRUG-CANDESARTAN_CILEXETIL, GENERIC_DRUG-TELMISARTAN, GENERIC_DRUG-LOSARTAN/HYDROCHLOROTHIAZIDE, GENERIC_DRUG-VALSARTAN/HYDROCHLOROTHIAZIDE, GENERIC_DRUG-OLMESARTAN/HYDROCHLOROTHIAZIDE, GENERIC_DRUG-IRBESARTAN/HYDROCHLOROTHIAZIDE, GENERIC_DRUG-CANDESARTAN/HYDROCHLOROTHIAZID, GENERIC_DRUG-TELMISARTAN/HYDROCHLOROTHIAZID, GENERIC_DRUG-VERAPAMIL_HCL, GENERIC_DRUG-AMLODIPINE_BESYLATE, GENERIC_DRUG-DILTIAZEM_HCL, GENERIC_DRUG-FELODIPINE, GENERIC_DRUG-NICARDIPINE_HCL, GENERIC_DRUG-CHLOROTHIAZIDE, GENERIC_DRUG-CHLOROTHIAZIDE_SODIUM, GENERIC_DRUG-HYDROCHLOROTHIAZIDE, GENERIC_DRUG-CHLORTHALIDONE, GENERIC_DRUG-METOLAZONE |
| NSAIDs | GENERIC_DRUG-DICLOFENAC_POTASSIUM, GENERIC_DRUG-DICLOFENAC_SODIUM, GENERIC_DRUG-DIFLUNISAL, GENERIC_DRUG-ETODOLAC, GENERIC_DRUG-FENOPROFEN_CALCIUM, GENERIC_DRUG-FLURBIPROFEN, GENERIC_DRUG-FLURBIPROFEN_SODIUM, GENERIC_DRUG-IBUPROFEN, GENERIC_DRUG-INDOMETHACIN, GENERIC_DRUG-KETOPROFEN, GENERIC_DRUG-KETOROLAC_TROMETHAMINE, GENERIC_DRUG-MEFENAMIC_ACID, GENERIC_DRUG-MELOXICAM, GENERIC_DRUG-MELOXICAM__SUBMICRONIZED, GENERIC_DRUG-KETOPROFEN__MICRONIZED, GENERIC_DRUG-INDOMETHACIN__SUBMICRONIZED, GENERIC_DRUG-NABUMETONE, GENERIC_DRUG-NAPROXEN, GENERIC_DRUG-NAPROXEN_SODIUM, GENERIC_DRUG-OXAPROZIN, GENERIC_DRUG-PIROXICAM, GENERIC_DRUG-SULINDAC, GENERIC_DRUG-TOLMETIN_SODIUM, GENERIC_DRUG-CELECOXIB |
| Corticosteroids | GENERIC_DRUG-HYDROCORTISONE, GENERIC_DRUG-DEXAMETHASONE, GENERIC_DRUG-DEXAMETHASONE_SODIUM_PHOSPHATE, GENERIC_DRUG-METHYLPREDNISOLONE, GENERIC_DRUG-METHYLPREDNISOLONE_ACETATE, GENERIC_DRUG-PREDNISOLONE, GENERIC_DRUG-PREDNISONE |
| Biologic Drugs | GENERIC_DRUG-DACLIZUMAB, GENERIC_DRUG-INTERFERON_BETA-1A, GENERIC_DRUG-INTERFERON_BETA-1B, GENERIC_DRUG-INTERFERON_BETA-1A/ALBUMIN, GENERIC_DRUG-NATALIZUMAB, GENERIC_DRUG-OCRELIZUMAB, GENERIC_DRUG-USTEKINUMAB, GENERIC_DRUG-VEDOLIZUMAB, GENERIC_DRUG-ADALIMUMAB, GENERIC_DRUG-INFLIXIMAB, GENERIC_DRUG-ETANERCEPT, GENERIC_DRUG-TOCILIZUMAB, GENERIC_DRUG-ANAKINRA, GENERIC_DRUG-RITUXIMAB, GENERIC_DRUG-ABATACEPT, GENERIC_DRUG-SECUKINUMAB |
| Immunomodulators | GENERIC_DRUG-DIMETHYL_FUMARATE, GENERIC_DRUG-FINGOLIMOD_HCL, GENERIC_DRUG-GLATIRAMER_ACETATE, GENERIC_DRUG-OZANIMOD_HYDROCHLORIDE, GENERIC_DRUG-PONESIMOD, GENERIC_DRUG-SIPONIMOD, GENERIC_DRUG-TERIFLUNOMIDE, GENERIC_DRUG-METHOTREXATE_SODIUM, GENERIC_DRUG-AZATHIOPRINE, GENERIC_DRUG-LEFLUNOMIDE, GENERIC_DRUG-SULFASALAZINE, GENERIC_DRUG-HYDROXYCHLOROQUINE_SULFATE, GENERIC_DRUG-TOFACITINIB_CITRATE, GENERIC_DRUG-BARICITINIB, GENERIC_DRUG-UPADACITINIB |
| Statins | GENERIC_DRUG-ATORVASTATIN_CALCIUM, GENERIC_DRUG-SIMVASTATIN, GENERIC_DRUG-ROSUVASTATIN_CALCIUM, GENERIC_DRUG-PRAVASTATIN_SODIUM, GENERIC_DRUG-LOVASTATIN, GENERIC_DRUG-FLUVASTATIN_SODIUM, GENERIC_DRUG-PITAVASTATIN_CALCIUM, DRUG-ATORVASTATIN_CALCIUM, DRUG-LOVASTATIN, DRUG-PRAVASTATIN_SODIUM, DRUG-SIMVASTATIN, DRUG-ROSUVASTATIN_CALCIUM, DRUG-FLUVASTATIN_SODIUM, DRUG-MEVACOR, DRUG-ALTOPREV, DRUG-LIPITOR, DRUG-PRAVACHOL, DRUG-ZOCOR, DRUG-CRESTOR, DRUG-LESCOL, DRUG-LESCOL_XL, DRUG-LIVALO |
| MHTs | DRUG-ALORA, DRUG-CENESTIN, DRUG-CLIMARA, DRUG-DELESTROGEN, DRUG-DIVIGEL, DRUG-ELESTRIN, DRUG-ENJUVIA, DRUG-ESTRACE, DRUG-ESTRADERM, DRUG-ESTRING, DRUG-EVAMIST, DRUG-FEMRING, DRUG-MENEST, DRUG-MENOSTAR, DRUG-MINIVELLE, DRUG-PREMARIN, DRUG-VIVELLE-DOT, DRUG-PROMETRIUM, DRUG-PROVERA, DRUG-ACTIVELLA, DRUG-ANGELIQ, DRUG-CLIMARA_PRO, DRUG-COMBIPATCH, DRUG-FEMHRT, DRUG-PREFEST, DRUG-PREMPRO |

| **Table S2. List of Diagnose Codes Used** | |  |
| --- | --- | --- |
| **Diagnosis** | **ICD-9** | **ICD-10** |
| Alzheimer’s disease | ICD-9-D-3310 | ICD-10-D-G300, ICD-10-D-G301, ICD-10-D-G308, ICD-10-D-G309 |
| Amyotrophic lateral sclerosis (ALS) | ICD-9-D-33520 | ICD-10-D-G1221 |
| Asthma | ICD-9-D-49300, ICD-9-D-49399 | ICD-10-D-J452, ICD-10-D-J45988 |
| Cardiovascular disease | ICD-9-D-4149, ICD-9-D-4280, ICD-9-D-4281, ICD-9-D-42820, ICD-9-D-42821, ICD-9-D-42822, ICD-9-D-42823, ICD-9-D-42830, ICD-9-D-42831, ICD-9-D-42832, ICD-9-42833, ICD-9-D-42840, ICD-9-D-42841, ICD-9-D-42842, ICD-9-D-42843, ICD-9-D-4289, ICD-9-D-4292 | ICD-10-D-I25700,ICD-10-D-I25708, ICD-10-D-I25709, ICD-10-D-I25710, ICD-10-D-I25719, ICD-10-D-I25720, ICD-10-D-I25729, ICD-10-D-I25739, ICD-10-D-I25798, ICD-10-D-I25799, ICD-10-D-I25810, ICD-10-D-I25811, ICD-10-D-I2582, ICD-10-D-I2583, ICD-10-D-I2584, ICD-10-D-I2589, ICD-10-D-I259, ICD-10-D-I501, ICD-10-D-I5043, ICD-10-D-I509 |
| COPD | ICD-9-D-49120, ICD-9-D-49121, ICD-9-D-49122, ICD-9-D-49320, ICD-9-D-49321, ICD-9-D-49322 | ICD-10-D-J440, ICD-10-D-J441, ICD-10-D-J449 |
| Chronic kidney disease | ICD-9-D-5851, ICD-9-D-5852, ICD-9-D-5853, ICD-9-D-5854, ICD-9-D-5855, ICD-9-D-5859 | ICD-10-D-N181, ICD-10-D-N182, ICD-10-D-N183, ICD-10-D-N184, ICD-10-D-N185, ICD-10-D-N189 |
| Congestive heart failure | ICD-9-D-7469, ICD-9-D-74689 | ICD-10-D-I5020, ICD-10-D-I5022, ICD-10-D-I5030, ICD-10-D-I5032, ICD-10-D-I5040, ICD-10-D-I5041, ICD-10-D-I5042 |
| Coronary artery disease | ICD-9-D-4110:ICD-9-D-4149 | ICD-10-D-I25:ICD-10-D-I259 |
| Type 2 Diabetes | ICD-9-D-25000, ICD-9-D-25001, ICD-9-D-25002, ICD-9-D-25003 | ICD-10-D-E108, ICD-10-D-E109, ICD-10-D-E1169, ICD-10-D-E118, ICD-10-D-E119 |
| Hypercholesterolemia | ICD-9-D-2720 | ICD-10-D-E780, ICD-10-D-E7800 |
| Hypertension | ICD-9-D-4010, ICD-9-D-4011, ICD-9-D-4019 | ICD-10-D-I10, ICD-10-D-I110, ICD-10-D-I119 |
| Ischemic heart disease | ICD-9-D-41000, ICD-9-D-41499 | ICD-10-D-I21, ICD-10-D-I229 |
| Multiple sclerosis | ICD-9-D-340 | ICD-10-D-G35 |
| Neurosurgery or brain cancer | ICD-9-D-01320, ICD-9-D-01321, ICD-9-D-01322, ICD-9-D-01323, ICD-9-D-01325, ICD-9-D-01326, ICD-9-D-01330, ICD-9-D-01333, ICD-9-D-1917, ICD-9-D-1918, ICD-9-D-1919, ICD-9-D-1983, ICD-9-D-2250, ICD-9-D-2375, ICD-9-D-2396, ICD-9-D-3481, ICD-9-D-3484, ICD-9-D-34882, ICD-9-D-7422, ICD-9-D-V1085, ICD-9-D-V1241 | ICD-10-D-A066, ICD-10-D-A1781, ICD-10-D-A5482, ICD-10-D-B431, ICD-10-D-C717, ICD-10-D-C718, ICD-10-D-C719, ICD-10-D-C7931, ICD-10-D-D330, ICD-10-D-D331, ICD-10-D-D332, ICD-10-D-D430, ICD-10-D-D431, ICD-10-D-D432, ICD-10-D-D496, ICD-10-D-G931, ICD-10-D-G935, ICD-10-D-G9382, ICD-10-D-S06317A, ICD-10-D-S06317S, ICD-10-D-S06327A, ICD-10-D-S06337A, ICD-10-D-S06337S, ICD-10-D-S06377A, ICD-10-D-S06380A, ICD-10-D-S06380D, ICD-10-D-S06380S, ICD-10-D-S06381A, ICD-10-D-S06381D, ICD-10-D-S06382S, ICD-10-D-S06384A, ICD-10-D-S06385S, ICD-10-D-S06387A, ICD-10-D-S06387S, ICD-10-D-S06389A, ICD-10-D-S06389D, ICD-10-D-S06389S, ICD-10-D-Z85841, ICD-10-D-Z86011 |
| Non-Alzheimer’s dementia | ICD-9-D-2900, ICD-9-D-29010, ICD-9-D-29011, ICD-9-D-29012, ICD-9-D-29013, ICD-9-D-29020, ICD-9-D-29021, ICD-9-D-2903, ICD-9-D-29040, ICD-9-D-29041, ICD-9-D-29042, ICD-9-D-29043, ICD-9-D-29410, ICD-9-D-29411, ICD-9-D-29420, ICD-9-D-29421, ICD-9-D-33119, ICD-9-D-33182 | ICD-10-D-F0150, ICD-10-D-F0151, ICD-10-D-F0280, ICD-10-D-F0281, ICD-10-D-F0390, ICD-10-D-F0391, ICD-10-D-G3109, ICD-10-D-G3183 |
| Obesity | ICD-9-D-2780, ICD-9-D-27800, ICD-9-D-27801, ICD-9-D-27802, ICD-9-D-27803 | ICD-10-D-E660:ICD-10-D-E669 |
| Osteoarthritis | ICD-9-D-71500, ICD-9-D-71599 | ICD-10-D-M1911, ICD-10-D-M1993 |
| Parkinson’s disease | ICD-9-D-332, ICD-9-D-3320 | ICD-10-D-G20, ICD-10-D-G214 |
| Pulmonary heart disease | ICD-9-D-4150:ICD-9-D-41799 | ICD-10-D-I26:ICD-10-D-I279 |
| Rheumatoid arthritis | ICD-9-D-7140, ICD-9-D-7142 | ICD-10-D-M0520:ICD-10-D-M061 |
| Stroke | ICD-9-D-430, ICD-9-D-431, ICD-9-D-432, ICD-9-D-4320, ICD-9-D-4321, ICD-9-D-4329, ICD-9-D-43300, ICD-9-D-4331, ICD-9-D-43310, ICD-9-D-43311, ICD-9-D-43320, ICD-9-D-43321, ICD-9-D-43330, ICD-9-D-43331, ICD-9-D-43381, ICD-9-D-43390, ICD-9-D-43391, ICD-9-D-43400, ICD-9-D-43401, ICD-9-D-43410, ICD-9-D-43411, ICD-9-D-43490, ICD-9-D-43491 | ICD-10-D-I6300, ICD-10-D-I63011, ICD-10-D-I63012, ICD-10-D-I63031, ICD-10-D-I63032, ICD-10-D-I6310, ICD-10-D-I63139, ICD-10-D-I63232, ICD-10-D-I63233, ICD-10-D-I63239, ICD-10-D-I63312, ICD-10-D-I6339, ICD-10-D-I6340, ICD-10-D-I63411, ICD-10-D-I63432, ICD-10-D-I6350, ICD-10-D-I63512, ICD-10-D-I63519, ICD-10-D-I63529, ICD-10-D-I6359, ICD-10-D-I638, ICD-10-D-I639 |
| Tobacco use | ICD-9-D-3051, ICD-9-D-98984, ICD-9-D-V1582 | ICD-10-D-F17220, ICD-10-D-F17221, ICD-10-D-F17223, ICD-10-D-F17228, ICD-10-D-F17229, ICD-10-D-F17290, ICD-10-D-F17291, ICD-10-D-F17293, ICD-10-D-F17298, ICD-10-D-F17299, ICD-10-D-Z720 |

| **Table S3. Incidence of Neurodegenerative Diseases at 1 and 3 Years After the Index Date** | | | | | | | | |  |  |  |  |  |  |
| --- | --- | --- | --- | --- | --- | --- | --- | --- | --- | --- | --- | --- | --- | --- |
| **INCIDENCE OF NEURODEGENERATIVE DISEASE 1 YEAR AFTER INDEX DATE** | | | | | | | | | | | | | | |
|  | total | NDD | NO NDD | AD | NO AD | DEMNOAD | NO DEMNOAD | MS | | NO MS | PD | NO PD | ALS | NO ALS |
| treat | 95154 | 6344 | 88810 | 1935 | 93219 | 3854 | 91300 | 27 | | 95127 | 831 | 94323 | 44 | 95110 |
| control | 95154 | 13544 | 81610 | 4789 | 90365 | 7551 | 87603 | 106 | | 95048 | 1940 | 93214 | 91 | 95063 |
| % treat |  | 6,67% | 93,33% | 2,03% | 97,97% | 4,05% | 95,95% | 0,03% | | 99,97% | 0,87% | 99,13% | 0,05% | 99,95% |
| % control |  | 14,23% | 85,77% | 5,03% | 94,97% | 7,94% | 92,06% | 0,11% | | 99,89% | 2,04% | 97,96% | 0,10% | 99,90% |
| **INCIDENCE OF NEURODEGENERATIVE DISEASE 3 YEARS AFTER INDEX DATE** | | | | | | | | | | | | | | |
|  | total | NDD | NO NDD | AD | NO AD | DEMNOAD | NO DEMNOAD | MS | | NO MS | PD | NO PD | ALS | NO ALS |
| treat | 95154 | 6190 | 88964 | 1876 | 93278 | 3748 | 91406 | 26 | | 95128 | 810 | 94344 | 41 | 95113 |
| control | 95154 | 12468 | 82686 | 4267 | 90887 | 6864 | 88290 | 76 | | 95078 | 1740 | 93414 | 89 | 95065 |
| % treat |  | 6,51% | 93,49% | 1,97% | 98,03% | 3,94% | 96,06% | 0,03% | | 99,97% | 0,85% | 99,15% | 0,04% | 99,96% |
| % control |  | 13,10% | 86,90% | 4,48% | 95,52% | 7,21% | 92,79% | 0,08% | | 99,92% | 1,83% | 98,17% | 0,09% | 99,91% |
